# Microglial Activation Modulated by P2X4R in Ischemia and Repercussions in Alzheimer’s Disease

**DOI:** 10.3389/fphys.2022.814999

**Published:** 2022-02-23

**Authors:** Carolina Castillo, Francisco Saez-Orellana, Pamela Andrea Godoy, Jorge Fuentealba

**Affiliations:** Laboratory of Screening of Neuroactive Compounds, Department of Physiology, School of Biological Sciences, Universidad de Concepción, Concepción, Chile

**Keywords:** P2X4R, microglial activation, ischemia, purinergic receptors, Alzheimer’s disease

## Abstract

There are over 80 million people currently living who have had a stroke. The ischemic injury in the brain starts a cascade of events that lead to neuronal death, inducing neurodegeneration which could lead to Alzheimer’s disease (AD). Cerebrovascular diseases have been suggested to contribute to AD neuropathological changes, including brain atrophy and accumulation of abnormal proteins such as amyloid beta (Aβ). In patients older than 60 years, the incidence of dementia a year after stroke was significantly increased. Nevertheless, the molecular links between stroke and dementia are not clearly understood but could be related to neuroinflammation. Considering that activated microglia has a central role, there are brain-resident innate immune cells and are about 10–15% of glial cells in the adult brain. Their phagocytic activity is essential for synaptic homeostasis in different areas, such as the hippocampus. These cells polarize into phenotypes or subtypes: the pro-inflammatory M1 phenotype, or the immunosuppressive M2 phenotype. Phenotype M1 is induced by classical activation, where microglia secrete a high level of pro- inflammatory factors which can cause damage to the surrounding neuronal cells. Otherwise, M2 phenotype is the major effector cell with the potential to counteract pro-inflammatory reactions and promote repair genes expression. Moreover, after the classical activation, an anti-inflammatory and a repair phase are initiated to achieve tissue homeostasis. Recently it has been described the concepts of homeostatic and reactive microglia and they had been related to major AD risk, linking to a multifunctional microglial response to Aβ plaques and pathophysiology markers related, such as intracellular increased calcium. The upregulation and increased activity of purinergic receptors activated by ADP/ATP, specially P2X4R, which has a high permeability to calcium and is mainly expressed in microglial cells, is observed in diseases related to neuroinflammation, such as neuropathic pain and stroke. Thus, P2X4R is associated with microglial activation. P2X4R activation drives microglia motility via the phosphatidylinositol-3-kinase (PI3K)/Akt pathway. Also, these receptors are involved in inflammatory-mediated prostaglandin E2 (PGE2) production and induce a secretion and increase the expression of BDNF and TNF-α which could be a link between pathologies related to aging and neuroinflammation.

## Introduction

Worldwide, there are over 80 million people currently living who have had a stroke (Lindsay et al., 2019). Over 13.7 million people have a stroke each year and 5.8 million dies because of it (Phipps and Cronin, 2020). Also, 116.4 million suffered permanent disabilities generated by stroke (Hachinski et al., 2019; Lindsay et al., 2019). It has been reported that 6 months post-stroke, around 35% of patients display depressive symptoms, 30% are unable to walk without assistance, and 26% are dependent on daily life activities (Lui and Nguyen, 2018). Stroke occurs when blood supply is interrupted, and there are two types: Ischemic and hemorrhagic (Vijayan et al., 2017). Ischemic strokes account for 85% of the total cases (Vijayan et al., 2017; Jayaraj et al., 2019; Lindsay et al., 2019). This type of stroke occurs when a blood vessel in the brain is occluded, causing a loss of the blood flow to an area of the brain (Randolph, 2016; Phipps and Cronin, 2020). The loss of blood flow causes the death of cells in the core of the injury, where the damage is irreversible, the surrounding area is called the penumbra and a loss of function is observed, but the damage is reversible (Phipps and Cronin, 2020). The ischemic injury in the brain starts a cascade of damaging cells events such as calcium dysregulation, increased reactive oxygen species (ROS) production, activation of caspases and calpain signaling to induce apoptosis and neuronal death, ultimately leading to neurodegeneration (Vijayan et al., 2017; Zhao et al., 2017; Castillo et al., 2018). The effects observed in patients depend on the brain region that is affected with supply oxygen and nutrients depletion (Vijayan et al., 2017). Due to this harmful process, some patients present neurological disorders. It has been estimated that stroke brings forward the onset of dementia by about 10 years (Vijayan et al., 2017; Lui and Nguyen, 2018). Current data estimates that dementia occurred in around 25% of people admitted to hospital with a stroke in the first year after this event (Hachinski et al., 2019). Furthermore, hazard ratios for dementia among patients who had strokes compared with the non-stroke population ranged from 2 to 8, in different studies of severe stroke cases (Sala Frigerio et al., 2019). Alzheimer’s Disease (AD) is the most common type of dementia, a neurological disorder characterized by neurodegeneration. From a biochemical perspective, AD is associated with synapse loss, synaptic dysfunction, mitochondrial structural and functional abnormalities, inflammatory responses, intracellular neurofibrillary tangles, and extracellular plaques (Trejo-Lopez et al., 2021). Clinically, AD is a multifactorial disease characterized by memory loss, multiple cognitive impairments, and progressive impairment of functional capacities. There are more than 100 pathological conditions that can be a risk factor for dementia, and stroke is the most common disease that can lead to this neurodegenerative disease (Vijayan and Reddy, 2016; Vijayan et al., 2017). AD and stroke have common risk factors, including hypertension, ROS increased levels, insulin resistance, type II diabetes, obesity, and hyperlipidemia (Hachinski et al., 2019; Sala Frigerio et al., 2019). Cerebrovascular diseases have been suggested to contribute to AD neuropathological changes including selective brain atrophy and abnormal accumulation of proteins such as amyloid beta peptide (Aβ), which is described as the main toxic factor in AD (Hachinski et al., 2019).

### Aging Related Diseases: Stroke Incidence and Alzheimer’s Disease

Research linking stroke and dementia have been focused on common vascular risk factors, ameliorated by lifestyle activities or medication, nevertheless one of the most important risk factors is aging (Vijayan and Reddy, 2016; Vijayan et al., 2017). The risk of having a first-time stroke increases exponentially from about 30 per 100,000 individuals at 30–39 years of age, to about 2000–3000 per 100,000 at ages above 85. Additionally, AD is mostly related to elderly patients, especially those over 80 years old (Mijajlović et al., 2017; Vijayan et al., 2017). Although, dementia due to stroke is not only aging-related, considering that this association has been reported in patients younger than 50 years old. Cognitive impairments occur in up to one-third of elderly patients with stroke (Lui and Nguyen, 2018). A mixed etiology of dementia and Vascular Dementia (VD) was thought to become more common with increasing age, but no clinical criteria for the diagnosis of dementia associated with stroke are available (Kluge et al., 2018). Stroke doubles the risk for dementia (post-stroke dementia), and approximately 30% of stroke patients develop cognitive dysfunction within 3 years (Kluge et al., 2018; Jayaraj et al., 2019; Lindsay et al., 2019). As a worrying statistic, stroke was the second cause of death and represented 9.1% of all deaths in 2017, and the third most common cause of deaths and disability combined (Delgado et al., 2010). It has been demonstrated that in patients older than 60 years, the incidence of dementia at 1 year after the stroke was 16%, while the prevalence was 22% (Lavados et al., 2005, 2021). The most frequent type of cognitive impairment was focal cognitive decline (Hachinski et al., 2019; Lavados et al., 2021). This study concludes that the risk of dementia after the first year in patients with severe strokes is very high, as it has been stated in several other studies (Lavados et al., 2021). Also, it has been mentioned the role of cerebrovascular disease, as a primary cause of cognitive impairment and to increase dementia risk caused by several factors, including AD or other neurodegenerative pathologies (Iadecola, 2013; Kalaria, 2018). During stroke and cerebrovascular diseases occurs hypoperfusion and blood brain barrer (BBB) disruption leading to oxidative stress and inducing tissue hypoxia by proteins extravasation. Hypoxia and oxidative stress activate pro-inflammatory pathways through kappa-light-chain-enhancer of activated B cells (NFkB) transcription, increasing cytokines and adhesion molecules in vascular cells, reactive astrocytes and activated microglia. That’s promote uncoupling in the neurovascular unit, contributing to damaged vascular cells (Iadecola, 2013).

Also, from a molecular perspective, a brain ischemia induces a cascade of pathophysiological processes, which increase brain ischemia and stimulates the inflammatory process, free radical production, excitotoxicity, disruption of sodium and calcium influx, enzymatic changes, endothelin release, delayed coagulation, activation of platelets and leukocytes, and endothelial dysfunction. Otherwise, dementia syndromes, such as AD, established after stroke were typically considered to be vascular in origin, and poststroke dementia might be the result of the effects of stroke and degenerative changes (Vijayan et al., 2017; Hachinski et al., 2019; Goulay et al., 2020). Nevertheless, the molecular links between stroke and dementia are not completely understood but are probably related to neuroinflammation (Kluge et al., 2018; Lui and Nguyen, 2018). Also, there are reported Longitudinal studies that have investigated the relation between inflammatory cytokines and post stroke dementia, suggesting interleukin 6, and interleukin 12 as predictors of post stroke cognitive impairment (Mijajlović et al., 2017).

Uncontrolled neuroinflammation, a hallmark of neurological disorders such as AD and stroke, may lead to neural dysfunction and aggravate disease progression. However, there are many questions raised in research linking stroke and dementia that are largely unanswered. Hence, it is important to understand early events of microglial cells activation, since they are the primary response involved in the inflammatory events during stroke or dementia such as AD.

### Role of Microglial Activation in Neuroinflammation

Microglia are brain-resident innate immune cells with myeloid origin. At the resting state they are constantly sensing the environment to detect injury (Zhao et al., 2017). They account for about 10–15% of glial cells in the adult brain and their phagocytic activity is essential for synaptic homeostasis in different areas, such as the hippocampus (Zhang et al., 2018; Lloyd et al., 2019). Microglial function is like peripheral macrophages and there has been described phenotypic changes against injury detection. Some authors divide microglia into a classical proinflammatory state (M1) and an alternative anti-inflammatory state (M2) (Wang et al., 2018). Microglia cells undergo an inflammatory polarization phenotype in response to injuries, such as pathogens or tissue damage. This phenotype is characterized by increased pro-inflammatory cytokines production, such as interleukin-1β (IL-1β) or tumor necrosis factor-α (TNF-α) and enhanced immune responses, including cellular recruitment through chemotaxis and phagocytosis (Subhramanyam et al., 2019; Leng and Edison, 2020). As a result, activated immune cells are recruited to the inflammatory site to remove the injury (Subhramanyam et al., 2019). However, this process must be tightly regulated because uncontrolled or excessive inflammation can lead to tissue damage (Baik et al., 2019). Microglial cells are the first line of defense, as they can destroy or eliminate different pathogens by secreting pro-inflammatory factors/cytotoxic mediators or through their phagocytic function (Zhang et al., 2018; Akhmetzyanova et al., 2019). Nevertheless, an extra accumulation of these mediators caused by microglia chronic activation can also improve neuronal damage and may increase post stroke symptoms (Wang et al., 2018). This statement has been confirmed by post-mortem analysis of brain chronic disorders, such as neurodegenerative diseases like AD (Forloni and Balducci, 2018; Sierksma et al., 2020). Microglia are involved in tissue repair, debris removal, and the maintenance of normal tissue dynamics after infection or injury, especially in the M2 polarized state. The resident microglia are M2 polarized during the early stages of stroke; however, they are transformed into the M1 polarized state in the ischemic penumbra region (Wang et al., 2018). Preclinical data indicate that anti-inflammatory therapy may be effective for stroke or AD, where the strategy is to modulate immune cell function by proinflammatory cytokine release suppression and enhancing anti-inflammatory cytokine expression (Sun et al., 2020).

### Phenotype M1 or M2 as an Indicator

Based on the injury and stimuli that induces microglial cells activation, these cells polarize into phenotypes or subtypes: the pro-inflammatory M1 phenotype, or the anti-inflammatory and immunosuppressive M2 phenotype (Forloni and Balducci, 2018; Song and Li, 2018; Lauro and Limatola, 2020). Phenotype M1 is induced by classical activation, where microglia secrete a high level of pro-inflammatory factors including Interleukin-1β (IL-1β), Interleukin-6 (IL- 6) and TNF-α, with high production of nitric oxide (NO) and ROS, which can cause damage to the surrounding neuronal cells (Hickman et al., 2018; Zhang, 2019), TNF-α can also induce an increase in the expression of glutaminase and Connexin-32, which increases the release of glutamate from microglia and enhances the excitotoxicity associated with neuronal damage (Takeuchi et al., 2006). Toll-like receptors (TLRs) have a fundamental role in innate immunity, and they can be activated by different molecules from pathogens called pathogen-associated molecular patterns (PAMPs) (Zolezzi and Inestrosa, 2017; Lauro and Limatola, 2020). This interaction between TLRs and PAMPs activates resident cells to release proinflammatory cytokines. TLR4 is highly expressed in microglia and TLR4-dependent microglial activation has been described on neurodegenerative diseases like AD or after stroke (Rojo et al., 2014; Tang and Le, 2015; Subhramanyam et al., 2019; Kolosowska et al., 2020). Additionally, TLR4 is also responsible for chronic neuroinflammation leading to brain damage after stroke, as it induces the production and release of TNF-α, IL-6, and NO, causing neuronal cell death (Bougarne et al., 2018; Song and Li, 2018; Lauro and Limatola, 2020). Otherwise, M2 phenotype is induced by a different mechanism of microglial activation (Liu et al., 2020). M2 microglia are the major effector cells with the potential to counteract pro-inflammatory reactions and promote repair genes expression. Moreover, after the classical activation, an anti-inflammatory and repair phase is initiated to achieve tissue homeostasis (Rojo et al., 2014). The anti-inflammatory cytokines IL-4, IL-13, IL-10, and TGF-β are the most secreted molecules by M2 subtype microglia to balance the pro-inflammatory responses. Thus, these activated cells secrete several factors for tissue repair and extracellular matrix remodeling (Zolezzi and Inestrosa, 2017). M2 polarization induces activation of the transcription factor (NFκB -p50) that is associated with the inhibition of M1- activation genes (Tang and Le, 2015). Secretion of IL-4, IL-10, and TGF-β by M2-activated microglia, promote innate immune responses, down-regulate M1-mediated reactions, and inhibit inflammatory functions. IL-10 is characterized as a cytokine that regulates pro-inflammatory response. Pre-treatments with IL-10 decrease nuclear translocation of the p50 and p65 subunits of NF-κB and production of several proinflammatory cytokines as IL-6 or TNF-α. Furthermore, IL-10 exerts neuroprotective effects and a diminished IL-10 level is associated with increased stroke risk (Sun et al., 2020). Nevertheless, the two microglia activated phenotypes mentioned, could transition into each other in different contexts that may contribute to pathogenic forms of neuroinflammation in chronic situations such as neurodegenerative diseases (Parada et al., 2015; Yang et al., 2017; Zhang et al., 2018; Castillo et al., 2019; Zhang, 2019; dos Santos et al., 2021).

In AD, microglia surrounding the plaques to phagocyte Aβ generally manifest M2 activation phenotype and this phagocytic activity of microglia is attenuated by pro-inflammatory cytokines such as IFN-γ, IL-1β, and TNF-α, which are mainly secreted by M1 microglia (Yang et al., 2017). Furthermore, the M2 phenotype is maintained at old ages of transgenic mice models, suggesting that activated microglia surrounding Aβ plaques adopted an alternative phenotype (Tang and Le, 2015). In 18-month-old mice, microglial activation is detected in hippocampal areas free of plaques, exhibiting M1 phenotypes that produce neurotoxic results (Hickman et al., 2018). Otherwise, after stroke, microglial cells are the first cell type to react, and they are activated by multiple cytokines and plasma proteins. Microglia adjacent to necrotic tissue and their peripheral regions at the beginning of a stroke are M2 type, phagocytizing and removing cell debris, necrotic tissue, and toxic metabolites (Hickman et al., 2018; Mancuso et al., 2019a,b). After approximately 24 h, M2 microglia are superseded by M1 subtypes, which release additional pro-inflammatory cytokines and exacerbate neuronal cell damage (Zhou et al., 2020). Finally, they release pro-inflammatory cytokines, chemokines, and neurotoxic factors, including IL-1β, IL-6, TNF-α, and NO that induce neurotoxicity (Wright et al., 2013; Wójtowicz et al., 2020). Related to this mechanism is the progression of many brain disorders, such as ischemic stroke and neurodegenerative diseases, where the microglia are chronically activated, amplifying the death of neurons. Also, studies have found that the expression of type M2 polarization receptors and IL-4 secretion decreased with age (Wang et al., 2018). Hence, suppression of the microglia-mediated neuroinflammation is a potential therapeutic strategy to treat these brain disorders or prevent the cognitive impairment associated with them. Recently the concepts of homeostatic and reactive microglia have been described and related to major AD risk factors (such as age and sex), linking to a multifunctional microglial response to Aβ plaques that evolves a continuous spectrum of microglial molecular phenotypes (Mancuso et al., 2019b; Sala Frigerio et al., 2019).

Besides, microglial activation into a pro-inflammatory or anti- inflammatory phenotype are driven by factors released from injured cells called damage-associated molecular patterns (DAMPs) or PAMPs, nonetheless, receptors and intracellular pathways involved are poorly understood. Several DAMPs are released in the brain during the inflammatory process such as misfolding proteins, nucleic acids, or nucleotides, mainly ATP (Di Virgilio and Sarti, 2018). Furthermore, the increase of extracellular ATP is recognized as a cell injury signal and pro-inflammatory stimulus. Microglial cells express receptors for extracellular ADP/ATP nucleotides denominated purinergic P2 receptors (P2Rs). There are two types of P2R: metabotropic P2YRs and ionotropic P2XRs (Suurväli et al., 2017).

### Association Between Purinergic Receptors and Microglial Activation

Purinergic receptors are activated by purines and are divided into two major families: the P1 or adenosine receptors and P2Y and P2X receptors activated by ADP/ATP (Burnstock, 2018). The first two types are G protein-coupled receptors, whereas P2X are ligand-gated ion channel receptors (Sáez-Orellana et al., 2015). To date eight P2Y receptors have been described in humans: P2Y1, P2Y2, P2Y4, P2Y6, P2Y11, P2Y12, P2Y13, and P2Y14 (Jacobson et al., 2020). These receptors are subdivided in two groups according to the primary G protein that they are associated with, P2Y1-like (P2Y1, 2, 4, 6, and 11) are coupled mainly with Gq, while P2Y12-like (P2Y12-14) are coupled with Gi (Jacobson et al., 2020). P2Y receptors are widely expressed in the CNS in all cell types, where they play an important role in glia-neuron communication, neurotransmission, and neurogenesis [for a more detailed review of P2Y in CNS please see: (Agostinho et al., 2020; Zarrinmayeh and Territo, 2020)].

In mammals, seven P2X subunits are described (P2X 1–7). The receptors are conformed by homo- or heterotrimers, with a central pore permeable to Na^+^, K^+^ and Ca^2+^ (Illes et al., 2021). Each subunit is composed of two transmembrane domains, intracellular N- and C-termini and a large extracellular loop. P2X subunits are widely expressed in CNS in all cell types, but some subunits are expressed only in certain cell types, such as P2X 1–3 in neurons and P2X 7 in glia (Illes et al., 2021). Of interest for us, P2X4R is expressed in neurons and glia, particularly in microglia; and is expressed in amygdala, basal ganglia, cerebellum, cerebral cortex, hindbrain, hippocampus, hypothalamus, midbrain, olfactory bulb, and spinal cord (Duveau et al., 2020). Native and recombinant P2X4R show a rapid activation and a slow and incomplete desensitization, and they have a high sensitivity to ATP (EC50 1-10μM) and permeability to Ca^2+^ (Kaiser et al., 2016). A particular characteristic of this purinergic receptor is its highly and constitutively internalization due to the presence of a non-canonic motif YXXGΦ which is mediated by the μ2 subunit of the adaptive protein 2 (AP2) and clathrin. This intracellular located P2X4R is functional and plays an important role in the secretion and activation of pulmonary surfactant (Fois et al., 2018), and in the fusion and trafficking of lysosomes (Murrell-Lagnado and Frick, 2019). It has been proposed that the homomeric receptors P2X 2, 4, and 7 dilate their central pore in response to prolonged stimulation, which could allow the permeation of molecules of up-to 800 Da, in the case of P2X 7 in microglia this transition pore would be able to induce cell death and apoptosis (Bernier et al., 2012). Some authors have argued that this process is not due to a pore dilation but rather due to the interaction with associated proteins, especially Pannexin-1 (Panx-1), considering that the use of inhibitors for this channel inhibited the permeability to large cations induced by prolonged ATP exposure (Pelegrin and Surprenant, 2006), however, newer evidence utilizing similar strategies have showed that even in the absence of Pannexin-1 by silencing or Knock out there is still permeation of larger molecules (Qu et al., 2011; Alberto et al., 2013). Some reports propose that there is an immediate permeation to large cations after ATP activation, for instance, single channel studies in P2X7R showed no increase in the amplitud or conductance in the channel during prolonged exposure to ATP (Raouf et al., 2007). More recently, it has been demonstrated that the P2X7R expression in purified lysosome (without any other cellular components) is sufficient to form channels permeable to molecules up to ∼900 Da and that lipid membrane constitution may play an important role in the P2X7R pore diameter (Karasawa et al., 2017). Therefore, the P2X7R solo expression seems sufficient to form a pore permeable to large cations upon immediate ATP exposure, nevertheless other mechanisms that allow these big molecules passage, such as Panx-1, are still possible.

The participation of different purinergic receptors in pathological processes has been described, among them the P2Y1, P2Y2, P2Y6, P2Y12, P2X4, and P2X7 receptor (Cieślak and Wojtczak, 2018). For many of these receptors, their upregulation and increase in their activity have been described. Interestingly the upregulation of P2X2, P2X4 and P2X7 has been reported in the brain of AD patients (McLarnon et al., 2006; Varma et al., 2009; Godoy et al., 2021). In addition, a recent report has shown P2X4R and P2X7R co-expression in human neurons from the frontal cortex with no differences between AD patients and age-matched control group, suggesting that the increased expression of P2XR in brain observed before, might be occurring in glial cells (Gaff et al., 2021).

The concomitant increase of P2X4R and P2X7R is interesting, because they share a high degree of sequence homology (45.3% in rat, 46.7% in human and 47.3% in mouse), and they are located in the same chromosome and in close proximity of each other in this organisms, and it is suggested that P2X7 arose as a gene duplication of P2X4 and therefore the mechanism of increased expression could be similar for both receptors (Loera-Valencia et al., 2015; Hou and Cao, 2016; Suurväli et al., 2017).

Furthermore, in mouse lung epithelial cells the silencing of P2X4R induces an increased P2X7R expression and, the P2X7R down-regulation improves P2X4R expression (Weinhold et al., 2010). In addition, it has been reported P2X4R negative mice with increased P2X7R expression with passenger mutations altering the receptor’s function (Er-Lukowiak et al., 2020; Ellegaard et al., 2021). Similar results were obtained using a transgenic mouse line that expresses soluble GFP by P2X7R promoter, increasing the expression of P2X4R (Ramírez-Fernández et al., 2020). However, has been reported a concomitant decrease in the expression of P2X4R and 7 in liver and kidney of mice deficient for P2X4R or 7 (Craigie et al., 2013; Besnard et al., 2016). Additionally, both receptors tend to be expressed in similar cell types suggesting that these receptors could have complementary functions and may be overlapped (Suurväli et al., 2017).

In spite of that, several results show an increment of ATP in the extracellular environment in different diseases such as AD and stroke (Wilkaniec et al., 2017; Ye et al., 2017; Srivastava et al., 2020). This extracellular ATP activates P2X receptors, increasing their activity and triggering toxic effects on neurons and glial cells. P2X receptor subunits are present in pre- and postsynaptic sites (Ye et al., 2017). Thus, presynaptic P2X receptors stimulate glutamate release in sensory neurons, whereby they may control intracellular calcium homeostasis. Notably, P2X4R is mainly expressed in microglial cells and is involved in several functions as pain control, anxiety, and memory (Bertin et al., 2020; Montilla et al., 2020; Inoue and Tsuda, 2021).

### Increased Expression of P2X4 Receptor on Microglial Cells

The first evidence of Purinergic receptors in ischemia was obtained in *in vitro* experiments by the group of Volontè in Italy. They tested chemically induced ischemia and hypoglycemia, together with wide-range P2 antagonists in cerebellar cultures, where they observed neuroprotection and a decrease in cell death (Cavaliere et al., 2001a,b). Importantly, they also described a marked increase of P2X4R expression (Cavaliere et al., 2002). Furthermore, they analyzed organotypic cultures and *in vivo* models of ischemia (carotid occlusion in gerbils) and using histological and biochemical analysis they observed an increase of P2X4R only in microglia (Cavaliere et al., 2003). Interestingly, *in vitro* the application of P2 agonists mimicked the effects of ischemia, and the use of non-selective antagonists decreased the cellular damage observed (Cavaliere et al., 2005). The increase of P2X4R in microglia is also observed in models of neuropathic pain (Tsuda et al., 2003), where the application of P2X antagonists and the silencing of P2X4R reduced the observed allodynia. This increased expression is also observed in other pathological processes, such as spinal cord injury, inflammatory pain, chronic migraine, and pre-term hypoxia-ischemia (Wixey et al., 2009; Toulme et al., 2010; de Rivero Vaccari et al., 2012; Tasca et al., 2015; Matsumura et al., 2016; Long et al., 2018; Su et al., 2019; Bertin et al., 2020; Trang et al., 2020). Otherwise, the overexpression of P2X4R in the hippocampus of a mice model altered Long Term Potentiation (LTP) and plasticity at CA1 synapses (Bertin et al., 2020). These findings may be related to an increased P2X4R expression in the hippocampus of AD patients with severe cognitive impairment, suggesting that upregulated P2X4R may contribute to synaptic dysfunction and microglia phagocytic function (Raouf et al., 2007; Godoy et al., 2019; Han et al., 2020; Nguyen et al., 2020; Trang et al., 2020).

The increased expression of P2X4R is mediated by sequential activation of Interferon Regulatory Factor 8 (IRF8), which is a critical regulator of reactive microglia, that induces the expression of repair genes including IRF5, that in turn binds to the promoter region of the *P2rx4* gene inducing its expression (Masuda et al., 2012, 2014). P2X4R up-regulation seems to be associated with the neuroinflammation process, which is also related to microglial activation (Raouf et al., 2007; Han et al., 2020). P2X4R activation drives microglia motility via the phosphatidylinositol-3-kinase (PI3K)/Akt pathway (Ohsawa et al., 2007). Also, these receptors are specifically involved in inflammatory-mediated prostaglandin E2 (PGE2) production, which contributes to pain-related inflammation (Montilla et al., 2020). The activation of this pathway by P2X4R increases the expression and induces the BDNF secretion by microglia (Ulmann et al., 2008; Trang et al., 2009; Tsuda et al., 2009). BDNF in response to ATP, induces a shift in the neuronal Chloride gradient, making GABA and Glycine less hyperpolarizing and in some cases depolarizing (Coull et al., 2005). This shift is produced via TrkB activation and a decrease in the expression of K^+^-Cl^–^ cotransporter KCC2 (Rivera et al., 2002; Ulmann et al., 2008; Trang et al., 2009). In AD models it has been observed a similar pattern, with decreased KCC2 expression, increased BDNF and TNF-α (Doshina et al., 2017; Zhou et al., 2021), which could link both pathologies related to aging and neuroinflammation.

Finally, during neuroinflammation, the activated glial cells secrete inflammatory mediators to modulate inflammatory responses. It has been described that the upregulation peak of P2X4R during ischemic injury in the brain occurs 2 days later and that its inhibition can promote the microglial polarization into a pro-inflammatory phenotype (Suurväli et al., 2017; Di Virgilio and Sarti, 2018). This evidence shows us the P2X4R regulation as a key point to inflammation process prevention. In addition to neurons and microglia, immunochemical studies have shown P2X4 receptor expression in astrocytes from hippocampal regions (Srivastava et al., 2020). More studies to determine their function and role in pathological processes such as neuroinflammation, are needed.

## Discussion

**FIGURE 1:**
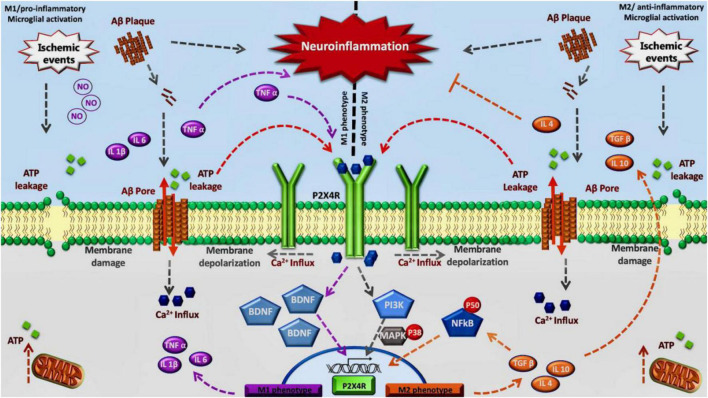
Inhibition of P2X4R in the acute phase could mitigate the effects of M1 transition allowing the appearance of the M2 phenotype: We propose this model considering that in the early stage P2X4 receptors contribute to maintain the M1 phenotype through TNF-α and BDNF signaling. Also, during a final stage, P2X4 may contribute to maintaining M2 phenotype by NFκB pathway.

Considering all this data, including the overexpression of P2X4R in diseases characterized by chronic neuroinflammation and activated microglial cells, the role of this purinergic receptor remains to be elucidated in humans. Some recent work using general KO mice and myeloid specific mice for P2X4R, together with a specific antagonist has shown that there is a critical time window, during the acute phase, where P2X4R inhibition is beneficial for the treatment of stroke, whereas a chronic inhibition could lead to an aggravated depressive state due to the lack of secretion of BDNF (Verma et al., 2017; Srivastava et al., 2020). Therefore, we propose an association between this receptor and the neuroinflammation progress observed in stroke and dementia. We postulate the modulation of P2X4R to regulate a specific change on M1/M2 phenotype of activated microglia observed in neuroinflammation associated with neurodegenerative diseases or stroke. Inhibition of P2X4R in the acute phase could mitigate the effects of M1 transition allowing the appearance of the M2 phenotype (Figure 1).

## Author Contributions

CC, FS-O, PAG, and JF contributed to the preparation, revision, and approval of the final manuscript. All authors contributed to the article and approved the submitted version.

## Conflict of Interest

The authors declare that the research was conducted in the absence of any commercial or financial relationships that could be construed as a potential conflict of interest.

## Publisher’s Note

All claims expressed in this article are solely those of the authors and do not necessarily represent those of their affiliated organizations, or those of the publisher, the editors and the reviewers. Any product that may be evaluated in this article, or claim that may be made by its manufacturer, is not guaranteed or endorsed by the publisher.
